# Curriculum Innovation: An Interactive, Case-Based, Multimodal Preclinical Neuroanatomy Teaching Curriculum

**DOI:** 10.1212/NE9.0000000000200308

**Published:** 2026-04-14

**Authors:** Cameron L. Hayes, Marinos G. Sotiropoulos, Galina Gheihman, Tamara B. Kaplan

**Affiliations:** 1 Department of Neurology, Mass General Brigham, Boston, MA; and; 2 Harvard Medical School, Boston, MA.

## Abstract

**Introduction and Problem Statement:**

Neuroanatomy is a core component of medical education yet may contribute to neurophobia, in part due to difficulty visualizing three-dimensional structures and applying foundational knowledge to clinical reasoning. Although active learning strategies such as flipped classrooms, case-based learning, and near-peer teaching are increasingly used to teach neuroanatomy, there is limited qualitative exploration describing how learners experience these modalities and how they support learning. This represents an important gap for educators designing learner-centered neuroanatomy instruction.

**Objectives:**

The aim of this curriculum was to enable preclinical medical students to (1) identify and describe major neuroanatomical structures, pathways, and vascular territories; (2) interpret basic neuroimaging to support anatomical localization; (3) distinguish central from peripheral causes of neurologic deficits; and (4) justify lesion localization by integrating clinical history, examination findings, and imaging.

**Methods and Curriculum Description:**

We evaluated a six-week preclinical neuroanatomy curriculum within the Harvard Medical School course Mind, Brain, and Behavior, which integrates asynchronous preparatory materials, in-person case-based collaborative learning (“mini-cases”), and hands-on cadaveric (“damp”) laboratory sessions facilitated by faculty and resident near-peer instructors. Learners completed required preparatory work before participating in paired in-person sessions emphasizing deliberate practice in anatomical localization. Program evaluation used mixed methods, including anonymous postcourse evaluation survey data and a semistructured focus group. Focus group transcripts were analyzed using a mixed deductive-inductive thematic approach.

**Results and Assessment Data:**

Of 168 enrolled students, 138 (82.1%) completed the postcourse evaluation. Overall course quality was rated as Excellent or Good by 94% (95% CI 89%–97%). Mini-cases and damp laboratory sessions were rated Excellent or Good by 94% (95% CI 85%–98%) and 74% (95% CI 62%–83%) of respondents, respectively. Twelve students participated in the focus group. Four major themes emerged: acquiring knowledge, applying knowledge, clinical relevance, and opportunities for improvement. Students highlighted the value of repeated case-based practice, near-peer clinical insight, and realistic exposure to neurology practice.

**Discussion and Lessons Learned:**

An integrated, multimodal neuroanatomy curriculum emphasizing active learning and clinical application was positively received and aligned with stated learning objectives. Key lessons include the importance of structured near-peer preparation, progressive scaffolding of complex anatomy, and repeated opportunities for application. These findings may inform the design of clinically oriented neuroanatomy curricula across institutions.

## Introduction and Problem Statement

Neuroanatomy is a fundamental component of medical education, as a core understanding of nervous system structures is necessary for localizing, diagnosing, and treating neurologic diseases. Neuroanatomy curricula vary widely among medical schools, often including a combination of lectures, laboratory sessions, and, more recently, flipped classroom and problem-based learning.^1^ While each of these methods has demonstrated educational benefits,^2-4^ the optimal integration of these modalities in teaching neuroanatomy remains uncertain.

*Neurophobia* is the fear of the neural sciences and clinical neurology arising from an inability to apply basic science knowledge to clinical situations.^5^ This phenomenon is believed to be engendered, at least in part, from students' perceptions of neurology as inherently more complex than other medical disciplines, with anatomy that is often difficult to visualize in 3D space.^6^ Other contributors include limited patient exposure, insufficient clinical teaching, and difficulty with performing the neurologic examination.^7^ Researchers have outlined evidence-based strategies for ameliorating neurophobia, including introduction of team-based learning, integration of 3D learning tools, and using standardized patients, among others.^8^ Despite these strategies, neurophobia remains prevalent: a 2024 meta-analysis estimated its global prevalence at 46% among medical students and young doctors.^9^ This highlights the need to better understand its causes, develop effective tools to combat it, and integrate these into medical school curricula.

In the “Mind, Brain, and Behavior” (MBB) preclinical neurosciences course at Harvard Medical School (HMS), we have introduced several innovations over the past decade to support active learning. In particular, the neuroanatomy component of MBB has undergone iterative development, incorporating specific design choices aimed at promoting clinical application and anatomical reasoning. These innovations include in-person case-based collaborative learning (CBCL) using short clinical vignettes (“mini-cases”), as well as hands-on damp laboratory anatomy sessions with near-peer resident instructors. Both of these were explicitly designed to allow students to participate in deliberate practice, the training of a specific skillset in an observed setting while receiving feedback in real time, which has been demonstrated to increase performance across various domains including medicine.^10,11^ Specifically, the mini-cases and laboratory sessions promote practice of lesion localization with immediate feedback from faculty and resident instructors. We, therefore, wished to further understand whether these recent curricular innovations have been effective, focusing on the student perspective.

In the past, postcourse evaluations of MBB have yielded largely positive feedback. However, these rely primarily on Likert-scale questions and provide limited depth into how and why specific teaching modalities are effective. We were interested in understanding students' in-depth experiences and perspectives on these teaching approaches as part of our evaluation of the curriculum. Evaluation is a critical stage of curriculum development, allowing educators to assess effectiveness through defined outcome measures.^12^ One method of evaluation includes qualitative or quantitative exploration of learners' perceptions, which offers insights into how curricular elements are experienced, integrated, and valued by learners. In our quality improvement effort, we triangulated focus group insights with data obtained from the postcourse evaluation to better assess how students experience these curricular elements and how they contribute to learning in preclinical neuroanatomy. Our aims were to understand *how* and *why* these teaching modalities worked for students in the course. Although situated within a single institution, this work was undertaken with the goal of generating insights that may inform the design and refinement of clinically oriented neuroanatomy curricula more broadly.

## Objectives

The following specific, measurable learning objectives were developed for our neuroanatomy curriculum for preclinical medical students. By the end of the course, students will be able to do the following:

1. Identify and describe the major neuroanatomical structures, pathways, and vascular territories of the central and peripheral nervous systems, including their core functional relationships.
2. Interpret basic neuroradiologic images (CT and MRI) to correlate anatomical abnormalities with clinical presentations.
3. Differentiate between central and peripheral causes of neurologic deficits.
4. Justify a proposed anatomical localization by integrating clinical history, examination findings, and imaging into a unifying explanation.

## Methods and Curriculum Description

### Overall Course Context

MBB is the 6-week preclinical neurosciences and neuroanatomy course at HMS that integrates foundational neuroscience, clinical neurology, neuroanatomy, and psychiatry. The course is designed to provide students with a clinically oriented framework for understanding disorders of the nervous system across disciplines, including neurology, psychiatry, neurosurgery, and others. Instructional methods include asynchronous preparatory materials, in-person case-based learning, patient-centered clinical experiences, and neuroanatomy laboratory sessions.

The in-person neuroscience and clinical content is distributed across the 6-week block, with neuroanatomy and neurology instruction occurring over 10 sessions spanning 5 weeks. Each session includes 2 hours dedicated to neuroanatomy and 2 hours dedicated to neurologic disease. Students complete assigned preparatory work before each session.

### Neuroanatomy Curriculum Structure

The neuroanatomy curriculum is intentionally designed to emphasize clinical application and anatomical localization. Foundational content is introduced before each session through curated textbook readings, concept videos, and a brief 15–20-minute recorded mini-lecture by course faculty. In addition, students are provided with access to supplemental materials such as *The Neurology Video Primer*,^13^ *Principles of Neural Science*,^14^ and *Merritt's Neurology*.^15^ The preparatory work allows in-person time to focus on application, spatial reasoning, and deliberate practice, with real-time feedback from instructors rather than didactic content delivery. This structure, known as the flipped classroom model, has been shown to enhance learner engagement and knowledge acquisition in health profession schools.^16,17^

Across 10 sessions, the neuroanatomy curriculum addresses major neuroanatomical systems, including overview of nervous system organization, corticospinal tract, somatosensory pathways and spinal cord, visual pathways, cerebral cortex and vascular territories, basal ganglia, cerebellum, brainstem and cranial nerves, extraocular movement and pupillary control pathways, and peripheral nervous system. Throughout all sessions, instruction is explicitly framed around how neuroanatomical knowledge is used clinically for lesion localization.

During each two-hour neuroanatomy session, students within each learning society are divided into 2 groups of approximately 20 learners. One group begins with faculty-facilitated localization mini-cases in a classroom setting for 1 hour. In these sessions, students work collaboratively through 4–6 brief clinical vignettes that require interpretation of symptom onset, neurologic examination findings, and neuroradiologic images to localize lesions. The sessions emphasize case-based learning in line with the predominant CBCL pedagogy at HMS.^18,19^ Faculty guide the discussion, emphasize reasoning strategies, and model expert clinical thinking.

The parallel group begins in the damp anatomy laboratory, where instruction is led by a core faculty member with support from 1 to 3 neurology, pediatric neurology, or neurosurgery residents or fellows serving as small-group instructors. Laboratory sessions incorporate hands-on interaction with cadaveric specimens, prosected materials, and three-dimensional anatomical models. Instruction alternates between guided exploration of relevant structures and pathways, structured review of spatial relationships, and correlation with cross-sectional imaging.

After 1 hour, groups switch so that all students experience both case-based and laboratory components. Faculty rotate roles across sessions, alternating between classroom-based facilitation and specimen-based instruction. This paired, multimodal structure is intentionally designed to integrate three-dimensional anatomy, imaging, and clinical reasoning, with repeated opportunities for students to apply neuroanatomical knowledge in clinically meaningful contexts.

### Assessment

Achievement of neuroanatomy knowledge is assessed through a midterm and final examination, both of which consist entirely of clinically oriented, vignette-based multiple-choice questions. In alignment with the learning objectives, they evaluate applied neuroanatomical reasoning rather than rote memorization.

Most questions require two-step reasoning: first identifying relevant neuroanatomical structures, pathways, or vascular territories and then applying principles of anatomical localization to interpret neurologic examination findings and neuroradiologic images. Examination items assess students' ability to localize lesions, distinguish central from peripheral pathology, correlate imaging with clinical presentation, and justify an anatomical localization through integrated use of history, examination, and imaging data.

### Postcourse Evaluation Survey

At the conclusion of MBB in August 2023, all students enrolled in the course were invited to complete an anonymous postcourse evaluation form to provide feedback for course leadership, as is done for all courses at HMS. This form contained a mix of multiple-choice and free-response questions about the course overall, specific elements of the curriculum, and experiences with faculty. The questions about specific elements of the curriculum were developed by the course director to gain insight into students' perceptions of different teaching modalities. Of note, these questions were developed before the conception of this project and were not influenced by findings from the focus group. Most questions were optional for form submission. Summary statistics for each question were calculated automatically as responses were collected.

### Focus Group

A semistructured focus group interview guide was developed de novo by the research team. A draft of the questions was informed by the postcourse evaluation data and the diverse perspectives of team members, which included a senior medical student (C.L.H.), a resident small-group instructor (M.G.S.), and the course director (T.K.). The draft guide was then reviewed by a member with expertise in qualitative research methods and experience moderating focus groups (G.G.). A final guide was determined by consensus and agreed on by the entire research team. The interview guide is available in eAppendix 1.

We used a convenience sampling approach to recruit students by email for the focus group. All 2023 MBB students who had recently completed the course were eligible to participate, and enrollment was offered on a first-come, first-serve basis. The 1-hour semistructured focus group was facilitated by an impartial third-party near-peer (C.L.H.) who was not involved with grading students, teaching, or course leadership. Before beginning the conversation, participants were assured that their comments would remain anonymous and confidential, and that participation was voluntary; all provided verbal consent. Students received a meal during the focus group and a $15 gift card incentive for participating. The focus group discussion was audio-recorded on 2 password-protected devices and transcribed using the online application Temi.^20^ Transcripts were manually reviewed, corrected for accuracy, and anonymized by C.L. Hayes before review by the entire team.

Qualitative analysis used a mixed deductive-inductive approach. Deductive codes were derived from focus group questions, while leaving room for inductive codes emerging from the data. An initial codebook was developed through iterative transcript review and refined through consensus by 3 researchers (C.L.H., M.G.S., G.G.).^21^ Two authors (C.L.H., M.G.S.) independently coded the transcript using qualitative software (Dedoose, SocioCultural Research Consultants, LLC, Los Angeles, CA).^22^ Any discrepancies were resolved through discussion and, where necessary, adjudicated by a third author (G.G.) familiar with the codebook.^21^ Final themes were identified and confirmed by consensus with the entire research team.^23^ While we were prepared to run multiple focus groups until data saturation, given a rich, in-depth discussion, we felt we had sufficient information power from 1 group to interpret findings from the survey in light of our qualitative results.^24^ The research team attended to reflexivity by being mindful of our roles as students, educators, and research investigators. C.L. Hayes was a medical student at the time of conducting the focus group and a student in the course 1 year before. M.G. Sotiropoulos was a resident near-peer tutor in the course and familiar with active teaching strategies used in the course. G. Gheihman was a former student in the course (before more recent changes in teaching strategies), a former near-peer educator in the course, and one of the current course faculty designing the interactive workshop. T.B. Kaplan was the course director responsible for curriculum changes; as such, she did not participate in coding or theme development. In their roles as educators, M.G. Sotiropoulos, G. Gheihman, and T.B. Kaplan are aware of adult learning theory principles and have used evidence-based learning sciences in the redesign of the curriculum. We acknowledge that our shared interest in improving medical student education and awareness of educational theory may have influenced interpretation. To account for this, we used an iterative codebook development and coding process, independently double-coded the transcript, and reached consensus on final themes to ensure that they reflected the data and not individual perspectives. At the same time, our previous experiences, perspectives, and positions in educational leadership allow us to implement student suggestions to improve the curriculum.

### Standard Protocol Approvals, Registrations, and Patient Consents

The HMS Office of Medical Education approved this project. Our proposal was deemed exempt from full IRB review as a curricular quality improvement initiative. Written consent was not required; verbal consent was obtained from participants at the beginning of the focus group.

### Data Availability

Anonymized data not published within this article can be made available by request from any qualified investigator.

## Results and Assessment Data

### Quantitative Analysis of Survey Data

In the MBB class of 2023, 138 of 168 students (82.1%) submitted a postcourse evaluation form. Overall course quality was rated as Excellent or Good by 94% of respondents (n = 130; 95% CI 89%–97%), while 6% rated the course as Average; no respondents rated the course as Fair or Poor.

Students were asked optional follow-up questions about the utility of the 2 neuroanatomy session types. Damp laboratory sessions with resident small-group instructors were rated as Excellent or Good by 74% of respondents (95% CI 62%–83%), while localization mini-cases were rated as Excellent or Good by 94% (95% CI 85%–98%). A detailed distribution of responses is presented in Table 1.

**Table 1:** Student Response Data From 2023 MBB Postcourse Evaluation Form

Question	n	Mean (SD)	Excellent %	Good %	Average %	Excellent + Good % (95% CI)
Please rate the course overall	138	1.6 (0.6)	50	44	6	94 (89–97)
Utility of damp laboratory sessions	69	1.9 (1.2)	55	19	13	74 (62–83)
Utility of mini-cases	66	1.4 (0.6)	67	27	6	94 (85–98)

Likert scale: 1 = excellent, 5 = poor. Confidence intervals calculated at the 95% level.

### Focus Group Thematic Analysis

Four major themes and 12 minor themes emerged from the focus group: (1) acquiring knowledge, (2) applying knowledge, (3) clinical relevance, and (4) course improvement. Students described acquiring knowledge through previous neuroscience exposure, MBB teaching sessions, and supplemental learning resources. Students applied their knowledge during damp laboratory sessions and mini-cases, and by answering probing questions from resident tutors. Clinical relevance was emphasized through clinical correlation by resident instructors, realistic case-based practice, and an end-of-course workshop showcasing neurology subspecialties. Areas for improvement included more 3D models, better resident tutor preparation, and more practice with covered content. Four representative quotes illustrating each of the major themes are provided in Table 2. The full selection of quotes (further divided into minor themes) from 10 of the 12 participants is available in eTable 1.

**Table 2:** Major and Minor Themes in MBB Neuroanatomy Focus Group Analysis With Representative Quotes

**Major theme** *Minor themes*	Representative quote
**Acquiring Knowledge** *Students' previous neuroscience experience* *In-person knowledge acquisition* *Supplemental learning resources*	“I actually had the cases [first] almost all the time, but I think I kind of appreciated like understanding the greater significance of things, and I think that the structure of the cases were much more like they teach you whereas like damp lab was much more like they kind of quiz you. And so at least from my perspective, it was nice to be taught it once and then when they came and asked us… what's happening here? What's this, what lesions would this represent? I kind of like had a basis to answer those questions.” (P12)
**Applying Knowledge** *Damp laboratory sessions* *Mini-cases* *Probing questions*	“I felt like it was a safe learning environment and I was totally fine with quizzing when it's related to the materials that we've been given.” (P1)
**Clinical Relevance** *Clinical correlation and peer advice by residents* *Case-based practice* *Reality of neurology practice*	“I'm not that interested in anatomy for the sake of anatomy, but I can get pretty excited about anatomy if I can apply it to like real things that happen to real people… I feel like if we were just pure anatomy, I would have a harder time like feeling motivated to apply myself as much.” (P1)
**Course Improvement** *More 3D virtual resources* *Better resident tutor preparation* *More practice with covered content*	“I thought when it came to like a lot of the neuroanatomy of… the ventricles and the sinuses and the basal ganglia, it was like really well done. We had like really good 3D dimensional discussion of it and then hands-on objects to look at to model it in the damp labs. But then I feel like when we got to the brainstem we didn't really have that and it was a lot more just like a picture and… here's the distribution of the arteries… and it was much harder to understand the 3D representation of the brainstem based on the instruction that we got in the table. And that might be because of the characteristics of the brainstem itself, but I think there's a lot of room for improvement there.” (P3)

#### I. Acquiring Knowledge

##### Students' Previous Neuroscience Experience

Some students had knowledge of the course from previous academic experiences in the neurosciences. This may have led to differences in preparedness for the new content within MBB.

##### In-Person Knowledge Acquisition

Students emphasized the importance of beginning each session with a strong knowledge foundation but differed on whether damp laboratory sessions or mini-cases best served this role. Some preferred starting with damp laboratory sessions to build foundational understanding before applying concepts in cases, while others felt mini-cases provided the initial framework needed to engage effectively in subsequent laboratory discussions.

##### Supplemental Learning Resources

Some students used supplemental resources outside the course, although most felt that these were not necessary for the examinations. Students noted variability in access to shared materials, as residents and faculty occasionally provided additional resources within individual academic societies.

#### II. Applying Knowledge

##### Laboratory Sessions

Anatomy laboratory sessions afforded a less structured, informal environment that allowed students to apply their knowledge through integration of anatomy learned in the preparatory material. Students felt that these sessions nicely complemented the more rapid-fire approach used in the mini-cases.

##### Mini-Cases

Students believed that the mini-cases were important for providing sufficient volume for the application of learned content. For instance, the cases helped emphasize the relevance of specific brain regions that were in the preparatory material.

##### Probing Questions

Resident tutors frequently asked impromptu questions to assess understanding. Students generally viewed this positively in a safe learning environment, although some found it less helpful, particularly when questions relied on the use of resources outside the MBB curriculum.

#### III. Clinical Relevance

##### Clinical Correlation and Peer Advice by Residents

Students valued resident instructors for highlighting clinically relevant content and clarifying what was most important for future practice, which supported more efficient learning.

However, variability in resident preparation and instruction across academic societies led to inconsistent learning experiences, particularly when residents were less familiar with session objectives or students' baseline knowledge.

##### Case-Based Practice

Students found value in the practice of applying their knowledge to realistic case presentations. Specifically, working with patient cases enhanced their motivation to learn neuroanatomy.

##### Reality of Neurology Practice

Students appreciated the end-of-course workshop where neurology subspecialists hosted hands-on interactive stations that showcased real-world neurology practice.

In addition, although MBB may have modified each student's interest in a career within neurology uniquely, students felt that the course clarified their understanding of neurology and supported informed career decision making, even when it did not increase interest in the specialty.

“I think that speaks to the strength of the course because… it comes with the breadth of the specialty and I feel like it was taught so well that I feel like I could really understand the thinking of a neurologist and like what it is that they have to do and sort of their line of logic when they're diagnosing something. And for me I was like, yeah, that's not what I like to do. That's not the way I like to think and not necessarily how I want to be doing that for the rest of my life. So I think it actually gave me a great idea of what [neurology] was and what it entailed, and I decided that it wasn't for me.” (P7)

#### IV. Course Improvement

##### More 3D Virtual Resources

Students reported difficulty visualizing complex 3D neuroanatomical structures and felt that greater use of virtual anatomy tools such as Visible Body^25^ would improve spatial understanding. These challenges were most pronounced for anatomically complex regions such as the brainstem.

##### Better Resident Tutor Preparation

Students felt that better resident tutor preparation, including standardization of structures to be covered, examination maneuvers to be taught, and materials to be used, would ensure that all students get the same valuable learning experience regardless of academic society or assigned group.

##### More Practice With Covered Content

Students indicated that more practice with course material, especially within topics such as neuroimaging and lesion localization, would help them translate newly learned anatomical knowledge into clinically useful skills.

## Discussion and Lessons Learned

By examining student perceptions of instructional approaches within an iteratively evolving neuroanatomy curriculum, this work contributes to understanding how specific design features of neuroanatomy education are experienced by learners and how they may support curricular goals. Neuroanatomy is a core component of preclinical neurology education that is taught in variable ways, and no single curriculum or teaching approach exists as a gold standard. Most medical students ranked the preclinical neuroscience course and its neuroanatomy components of damp laboratory sessions and mini-cases highly. In our thematic analysis, we identified 4 major themes describing students' perspectives and experiences learning in the course: (1) acquiring knowledge, (2) applying knowledge, (3) clinical relevance, and (4) suggestions for course improvement. It is important to note that the identified themes were found to align with the intended learning outcomes, particularly relating to anatomical localization, application of knowledge to clinical scenarios, and integration of imaging with examination findings. Students' emphasis on the importance of repeated practice, clinical relevance, and multimodal instruction reflects the cognitive demands inherent in achieving these objectives.

To place our findings within established educational theory, we interpreted the emergent themes through Knowles theory of andragogy, a foundational model of adult learning.^26^ Knowles outlined 4 core principles of effective adult learning: learner involvement in instruction, integration of new knowledge into previous knowledge, relevance, and problem centeredness.^26^

The first principle emphasizes active learner engagement throughout the instructional process. MBB is intentionally designed to support this through flipped-classroom learning. Consistent with this principle, focus group participants offered thoughtful suggestions for course improvement and described independently seeking supplemental resources, reflecting a self-directed approach to learning and alignment of study strategies with individual learning preferences.

The second principle in adult learning emphasizes integration of new knowledge with previous experience. In accordance with this, a prominent theme in our focus group was knowledge acquisition shaped by learners' varied exposure to the neurosciences. Students felt that experiences in the neurosciences facilitated learning in MBB, highlighting differences in foundational knowledge. Despite the variability, students unanimously agreed that beginning each session with foundational teaching was essential for subsequent application, although they differed on whether the damp laboratory or the mini-cases session best served this role.

Third, Knowles posited that adult learners benefit from instruction that is immediately relevant to them. In our study, we found that a key theme of clinical relevance emerged from the focus group discussion. Students emphasized the value of resident teaching assistants, noting their essential role in linking course content with practical clinical experience from the wards. They also highly valued the end-of-course interactive workshop, where rotating through neurology subspecialty stations provided a more realistic view of daily neurologic practice.

Finally, Knowles proposed that effective adult learning should be problem-oriented rather than content-oriented. To this end, MBB uses an active learning approach throughout its curriculum. Focus group participants agreed that active engagement in solving patient cases was essential for applying their newly acquired knowledge and for motivating further study. Specifically, students lauded the mini-cases for the opportunity to practice problem-based learning and the damp laboratory sessions for their effective integration of coursework. Moreover, students appreciated practicing active recall through answering probing questions from resident tutors.

Near-peer instruction from resident tutors is a core aspect of the course. While near-peer instructors were generally well received by students, some variability in the level of preparation and teaching quality was noted. Residents in the damp laboratory were able to “[keep] it real” (P7) by explaining which concepts learned during the course were most clinically relevant. This has previously been noted as one of the benefits of near-peer education; peer teachers are able to more easily distinguish between “essential knowledge” and “minutiae” for learners.^27^ Moreover, medical students tend to judge near-peer teachers as positive contributors to their learning of neuroanatomy, and better-trained near-peer educators are associated with significantly improved perceptions of neuroanatomy understanding, motivation to learn neuroanatomy, and greater safety of the learning environment.^28^ Our qualitative results highlight a tension between near-peer approachability and perceived variability in instructional quality that may be associated with residents' individual preparation. Informed by this finding, neuroanatomy laboratory sessions with near-peer teachers should incorporate structured resident preparation, including instruction on learning objectives for each session and on effective teaching strategies; this is an actionable area of improvement for our course.

Our qualitative findings help explain why students rated the quality of instruction highly and clarify which aspects of mini-cases and damp laboratory sessions most supported learning. It is important to note these insights extend beyond our course and may inform instructional design in other neuroanatomy curricula. The themes we identified align with Knowles theory of andragogy and suggest concrete evidence-informed strategies for neuroanatomy education. Consistent with previous studies, these include activation of previous knowledge constructs through review,^29^ self-directed exploration (e.g., modules and supplemental resources),^30,31^ integration of numerous teaching modalities within each session,^31^ opportunities to apply knowledge through problem-based learning,^29,31^ and explicit links between anatomical content and its clinical relevance to enhance motivation and engagement.^31-34^ We also identified suggestions for improving our course, including better integration of visual models and neuroimaging, as well as more consolidation opportunities. Table 3 presents actionable recommendations for educators designing or refining neuroanatomy curricula that emphasize clinical reasoning and anatomical localization.

**Table 3:** Actionable Recommendations for Neuroanatomy Course Improvement

Recommendation	Explanation
Implement structured training and resource packages for resident facilitators	Promotes consistency in teaching quality across small groups while preserving the benefits of near-peer instruction and reducing variability in learner experience
Scaffold 3D neuroanatomy content progressively across the course	Introduces complex 3D structures in a staged manner, supporting incremental knowledge building and reducing cognitive load. Combining static images with dynamic visualization tools may further enhance spatial understanding
Expand clinical exposure through longitudinal cases across multiple sessions	Reinforces clinical relevance of anatomy and deepens understanding by linking new concepts to previous learning
Use outcomes-based design of in-person sessions, aligning prep materials with active learning	Ensures prep work, laboratory sessions, and case discussions target shared learning objectives optimizing retention and engagement
Invest in workshops for faculty/residents on adult learning principles (e.g., andragogy, cognitive load theory, and retrieval practice)	Establishes a shared pedagogical framework and supports consistent use of evidence-based teaching strategies

A strength of our study is the high response rate to the postcourse evaluation, representing perspectives from almost all students. The focus group was confidential and anonymous, allowing learners to share candid perspectives. The presence of both positive feedback and constructive criticism further supports the credibility and balance of the data. There are also several limitations. First, our data reflect a single academic year and one focus group conducted at a single institution at a single time point. This was in part due to time limitations, as the student population interviewed was nearing the start of their core clerkships, which limited the feasibility of recruiting participants for additional focus groups. Despite this, we felt the focus provided sufficient depth and breadth of information to support analysis and discussion of the findings. There may have been selection bias: students who chose to participate in the focus group may have held more positive or negative perspectives on the course. The quantitative survey was part of regular course evaluations and not created, piloted, or validated for the purpose of this curricular quality improvement work. These results may not generalize to the broader medical student population or to other institutions with different curricular structures.

Our findings provide new insights into how preclinical students experience a multimodal neuroanatomy curriculum and highlight instructional strategies that can better support learning. Evidence-based strategies such as active learning, clinical integration, and learner-centered approaches can strengthen students' confidence and fluency in neurology. Future work should refine and evaluate instructional approaches that reduce neurophobia and prepare the next generation of physicians for high-quality neurologic care.

## Glossary

CBCL: case-based collaborative learning
HMS: Harvard Medical School
MBB: Mind, Brain, and Behavior
